# Supplementary material for: Mapping the chaperonin TRiC/CCT interactome in mouse photoreceptors reveals functional significance for energy metabolism
Source: PLoS One. 2026 Jul 29;21(7):e0345843. doi: 10.1371/journal.pone.0345843 (PMC13419182; doi:10.1371/journal.pone.0345843)
Supplement: S1 File — (PDF) [file pone.0345843.s008.pdf]

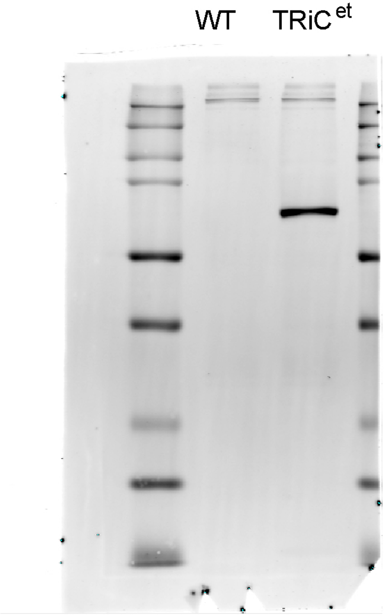

Fig1B

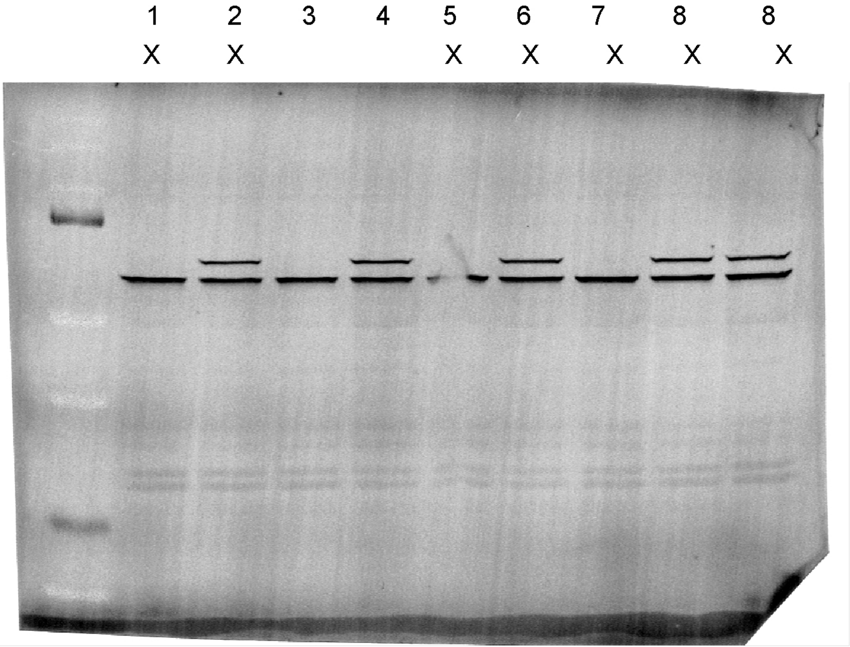

Fig1C

1,3,5,7: WT
2,4,6,8: TRiC^{et}

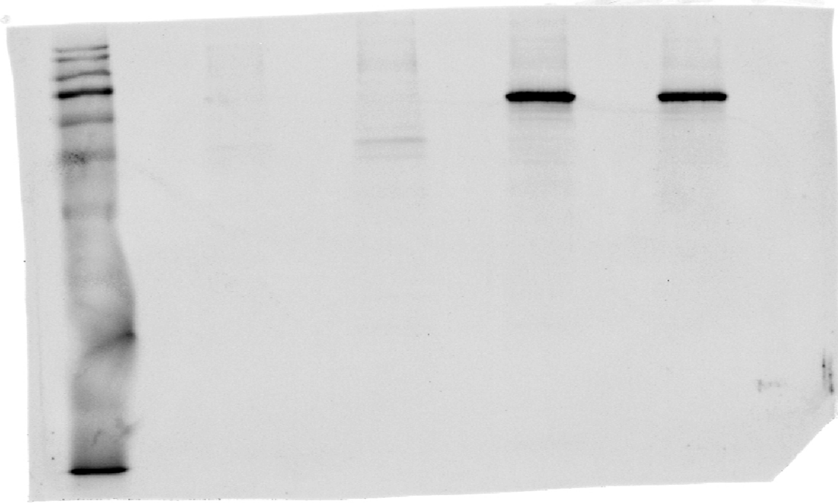

Fig3B Left, top (WB: anti-FLAG)
see lanes annotaion in Fig3B

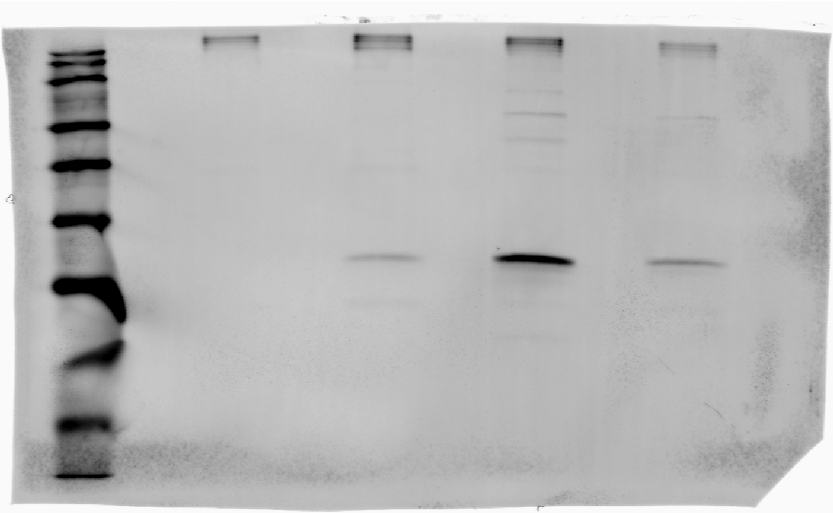

Fig3B, Left, bottom (WB: anti-His-tag)
see lane annotation on Fig3B

WB: anti-FLAG WB: anti-His WB: anti-tcp-1β

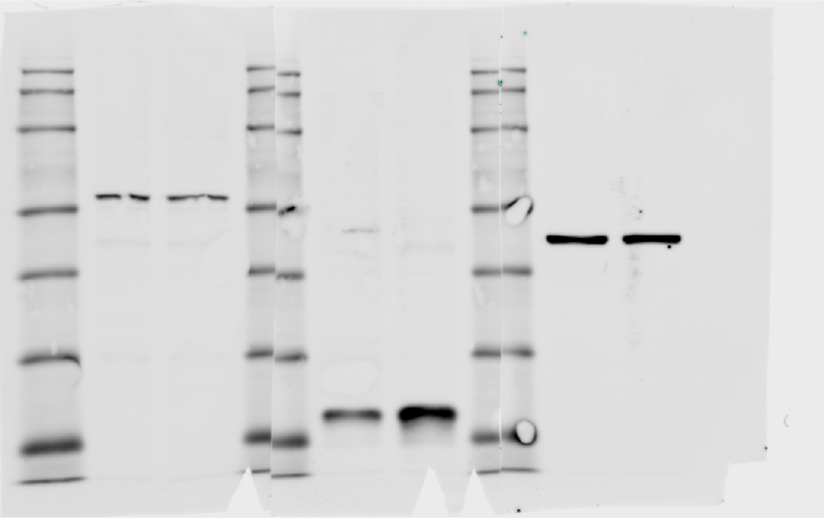

Fig3B, Center

see lane annotation on Fig3B

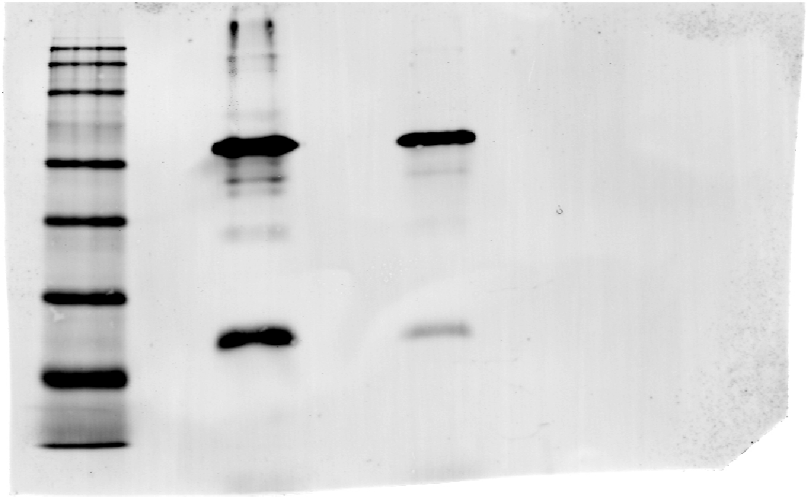

Fig3B, Right (WB: anti-FLAG + anti-His tag)
see lane annotation on Fig3B

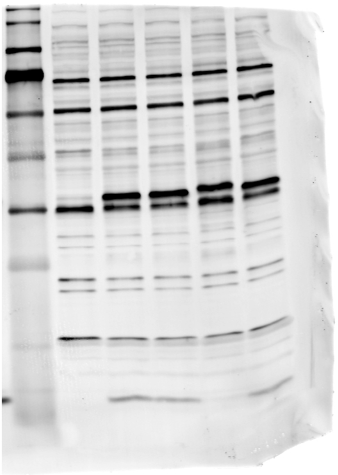

Fig9B, Left
WB: anti-myc + anti-Gβ1 + anti-HA
see lane annotation in Fig9B

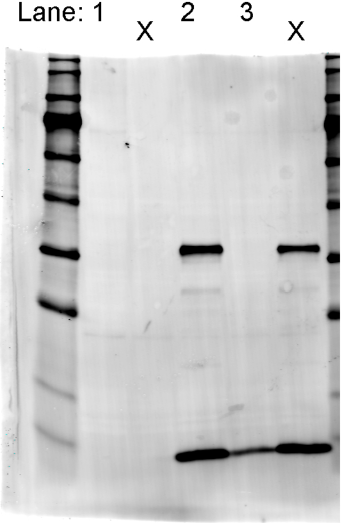

Fig 9B Right
WB: anti-myc + anti-HA
see lane annotation on Fig9B

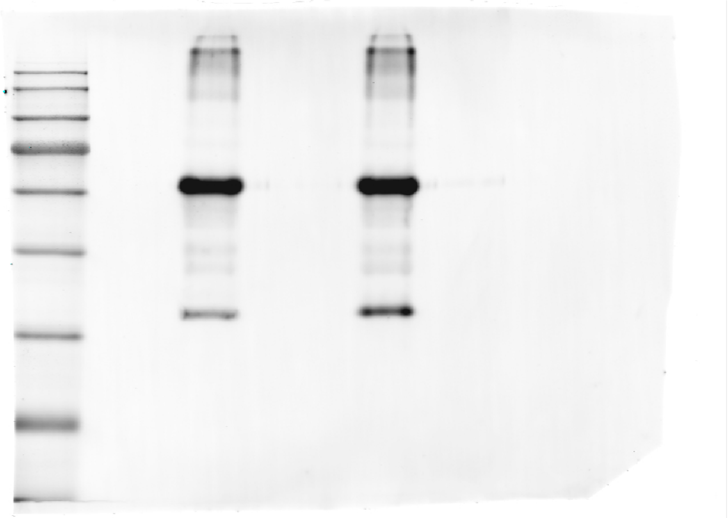

Fig9C (WB: anti-tcp-1γ + anti-myc)
See lane annotation on Fig 9C

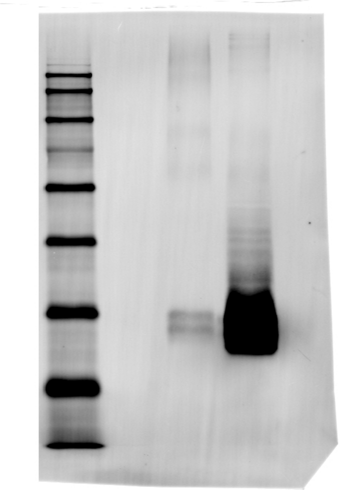

Fig9F Left (WB: anti-myc)
see lane annotation on Fig9F

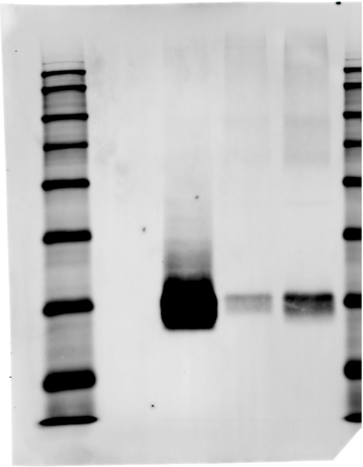

Fig9F, Right (WB: anti-myc)
see lane annotation on Fig9F
